# One SMS a day keeps the stress away? A just‐in‐time planning intervention to reduce occupational stress among apprentices

**DOI:** 10.1111/aphw.12340

**Published:** 2022-01-20

**Authors:** Konstantin Schenkel, Severin Haug, Raquel Paz Castro, Janina Lüscher, Urte Scholz, Michael P. Schaub, Theda Radtke

**Affiliations:** ^1^ Department of Psychology, Social and Health Psychology University of Zurich Zurich Switzerland; ^2^ Swiss Research Institute for Public Health and Addiction University of Zurich Zurich Switzerland; ^3^ Department of Psychology, Social and Health Psychology, University Research Priority Area Dynamics of Healthy Aging University of Zurich Zurich Switzerland; ^4^ Health Psychology and Applied Diagnostic, Institute of Psychology University of Wuppertal Wuppertal Germany

**Keywords:** apprentices, just‐in‐time planning intervention, multilevel modeling, occupational stress

## Abstract

**Background:** Occupational stress is one of the main sources of stress in apprentices with physical and psychological health consequences. Just‐in‐time planning interventions (JITPIs) are one opportunity to deliver intervention components at the right times and locations to optimally support apprentices in stressful situations. The aim of this study was to test the proximal effect of a mobile phone‐delivered JITPI to reduce occupational stress in 386 apprentices within a planning intervention.

**Methods:** An AB/BA crossover design in which participants were randomly allocated to (A) the planning intervention or (B) the assessment only condition was implemented.

**Results:** The analyses of the study “ready4life”, multilevel modeling, revealed no significant effect of the planning intervention on occupational stress reduction.

**Conclusions:** Possible reasons for the missing effect might be the low stress level of participants or the type of the intervention delivery. Since apprenticeships in Switzerland differ considerably, future studies should enable more adapted interventions for the apprentices and consider individual circumstances of stress. Further, the intervention should focus on apprentices with high occupational stress levels or a high‐risk of stress. Studies should investigate exactly when and why a person needs support regarding her/his occupational stress. Therefore, objective measurements of stress could be helpful.

## INTRODUCTION

For the last decades, an increasing number of adolescents and young adults are experiencing stress (American Psychology Association, 2018). Work and apprenticeship are main causes for adolescents and young adults feeling fundamentally stressed (American Psychology Association [APA], 2015; Güntzer, 2017). Occupational stress is defined as “a physiological and psychological response to events or conditions in the workplace that is detrimental to health and well‐being*…*” (APA, 2021). In Switzerland, employees and apprentices under 30 years are increasingly confronted with occupational stress. In 2012, 18% of young adults experiences occupational stress with an increase to 21% in 2017 (Federal Statistical Office, 2019). Similar data have also been reported from other industrial countries in the western hemisphere (APA, 2015). Reasons for occupational stress might be time and work pressure, concentration demands, job insecurity and work interruptions (Grebner et al., 2005).

Regarding occupational stress of adolescents and young adults in Switzerland it is important to mention that a majority has a vocational education (Fazekas & Field, 2013). The Swiss education system is differentiated between a vocational training and academic training at a college/university. An apprenticeship is a key element of the education system and is the main prerequisite to many educations (e.g., nursing). During these 3 years of education program, the apprentices have to accomplish a vocational training at school and a simultaneous apprenticeship in a company. The apprentices usually attend vocational school 2 days a week and work at the workplace for 3 days (Educationsuisse, 2021). Therefore, the apprentices have a similar workload as compared with employees. Consequently, 42% of 16‐ to 24‐year‐old Swiss employees and apprentices are in a critical stage in the Job Stress Index (Gesundheitsförderung Schweiz, 2020). This is explicable by the fact that apprentices face an additional challenge in balancing vocational training and job‐related requirements (Lang et al., 2017).

Possible consequences of such high stress levels exist for the physical (i.e., headaches and dizziness) and the mental health (i.e., fatigue and exhaustion; Ottová‐Jordan et al., 2015). Prospective studies indicated that stressors during adolescence and young adulthood predict an increase in psychopathological symptoms (Bob et al., 2013). Other consequences relating with (occupational) stress are reduced well‐being (van Loon et al., 2020), less frequent physical exercises, worse nutrition, and poorer sleep (Åkerstedt et al., 2007; Ng & Jeffery, 2003). In addition, people experiencing occupational stress are more exhausted and show less vigor resulting in low work performance and job burnout in comparison with people without occupational stress (Demerouti & Bakker, 2011; Jonsdottir et al., 2013; Mäkikangas et al., 2014; Syrek et al., 2013). Research also showed that the level of work‐related experiences like stress or recovery depends upon energetic resources like vigor (Sonnentag & Niessen, 2008). Further, findings suggest that exhausted employees find it more difficult to detach and recover from stress at work—although they need it most (Sonnentag et al., 2014). Therefore, it is important to examine vigor and exhaustion as possible moderators that might impact the effect of an intervention to reduce perceived occupational stress (Mäkikangas et al., 2014; Sonnentag & Niessen, 2008; Syrek et al., 2013). Thus, future research should examine whether apprentices and employees with higher levels of exhaustion and lower levels of vigor benefit from an intervention to decrease stress at the workplace/apprenticeship site to a larger extent compared with those individuals who report lower exhaustion and higher vigor.

Due to the increased stress levels among Swiss adolescents and young adults plus the resulting negative health consequences, more research is necessary to prevent perceived occupational stress of apprentices. The meta‐analysis by Yusufov et al. (2019) indicated that interventions to reduce perceived stress in the fields of academic pressures, social challenges, family strains, and financial concerns from undergraduate and graduate students have a moderate overall effect on stress. However, these analyzed interventions were often very complex (e.g., several intervention modules) and time‐consuming (e.g., participation in a stress reduction course lasting several weeks) for the participants (Yusufov et al., 2019). Therefore, a health behavior change intervention program with little effort for the participants to reduce perceived occupational stress during the apprenticeship is needed. One advantage of this approach would be to offer the intervention at appropriate times or contexts, in order to support participants in critical situations and to reduce participants' burden (Smyth & Heron, 2016).

One of the most promising behavior change techniques (BCT) is “planning” (Hagger & Luszczynska, 2014; Michie et al., 2013). An important planning intervention technique is “if‐then‐plans” (BCT 1.4; Hagger & Luszczynska, 2014; Michie et al., 2013). When using this BCT, an individual associates a situational cue (when/where) to an intended behavioual response (how) by mentally simulating the expected situation (Gollwitzer, 1999). This establishes a link between a specific cue and an intended action to translate goal intentions into behavior. For instance, “If situation Y is encountered, then I will initiate the goal directed behavior X!”. This strategy has medium to large effect sizes on behavior observed across various populations, modes of delivery (e.g., internet‐ or laboratory based interventions) and behaviors and has been confirmed as successful in the context of stress reduction in general (Gollwitzer, 1999; Gollwitzer & Sheeran, 2006; Hagger & Luszczynska, 2014) as well as concerning work‐related stress (Gollwitzer et al., 2018). Further, research indicates that one‐time performed planning interventions are effective for behavior changes (Hagger & Luszczynska, 2014). Therefore, planning could be a fast proactive technique with little effort to reduce the perceived stress during a stress event at the apprenticeship site (Hagger & Luszczynska, 2014). In contrast to the traditional mode of delivery of planning interventions (Hagger & Luszczynska, 2014) just‐in‐time planning interventions (JITPIs) have the possibility to be more easily tailored to everyday life during times and in places or situations when individuals may be most susceptible (Hardeman et al., 2019; Nahum‐Shani et al., 2018).

JITPIs have the potential to address situations in which people are likely to engage in unhealthy behaviors or experience negative consequences of their behavior (Hardeman et al., 2019). They can create opportunities for supportive interventions “in the moment” of unhealthy behaviors or negative consequences (Hardeman et al., 2019). To receive a JITPI, however, the individual must be receptive; that is, he or she should not be confronted with other distracting tasks (e.g., school lessons; Nahum‐Shani et al., 2015). The general aim of JITPIs is to improve the access to health behavior change interventions via mobile devices such as mobile phones (Hardeman et al., 2019). The published studies on the effects of JITPIs are promising, because they indicate that JITPIs give a direct and a system‐triggered behavioral support corresponding to a need in the right‐time (Hardeman et al., 2019; Haug et al., 2020). So far, the effectiveness of JITPIs has not been addressed for occupational stress, but conceptually JITPIs can also be applied to occupational stress reduction, since the mechanisms behind are independently of the target behavior (Hardeman et al., 2019). Particularly, it is assumed that by sending a JITPI at short notice before a possible stressful situation, the plans will be recalled in the stressful situation and that consequently, the planned strategy to reduce perceived occupational stress will be applied. Therefore, the aim of this study was to examine within a within‐design the effects of a text message‐based JITPI on perceived occupational stress reduction at the workplace among apprentices. For the present study, it was hypothesized that (1) on days when apprentices receive the just‐in time delivered planning intervention, apprentices report lower levels of perceived occupational stress during vocational training compared with days without the just‐in time delivered planning intervention. It is further postulated that (2) apprentices with lower levels of work‐related vigor will benefit from the just‐in‐time delivered planning intervention to a higher degree compared with apprentices with higher levels of work‐related vigor. Additionally, it is assumed that (3) apprentices with higher levels of work‐related exhaustion will benefit from the just‐in‐time delivered planning intervention to a higher extend than apprentices with lower levels of work‐related exhaustion.

## METHODS

### Design

In 2017 and 2018, apprentices aged 16 years or older were recruited in Switzerland by vocational and upper secondary schools plus companies participating in the comprehensive life skills program “ready4life” (Haug et al., 2017). This project consisted of two independent modules, each lasting 4 months. Module 1 focussed on self‐competencies, in particular dealing with stress and emotions with the aim to handle apprentice's perceive stress level in stressful situations. Module 2 focussed on health in general and resistance to the abuse of legal and illegal substances. Both modules used short message services (SMS) to deliver the intervention. For more details, see Haug et al. (2017). The JITPI to reduce perceived occupational stress among apprentices that is tested for its effectiveness in this paper took place in module 1.

The JITPI contained an AB/BA crossover design meaning that every participant received a planning intervention (A) and a control condition (B) in a randomized order. Each condition was performed only once. Prior to the JITPI, apprentices filled‐in a baseline assessment. The baseline assessment took place during a regular school lesson on health education. Participants provided their sociodemographic data and information about the study's relevant variables (e.g., vigor). Apprentices indicated their perceived stress level at the apprenticeship site. Apprentices with at least a medium perceived stress level were then presented with nine if‐then plans (BCT 1.4; Michie et al., 2013). Each if‐then plan included the stem “If I'm stressed out at my apprenticeship site, then …” followed by strategies like ¨positive reframing (e.g., … I think about the positive aspects of the situation [e.g., new experiences]), distraction (e.g., … I take a short break), control strategies (e.g., *…* I tell my teacher or work colleagues that I need help), self‐efficacy boost/think of advantages (e.g., *…* I think of a similar situation that I have successfully mastered at my apprenticeship site), or active coping (e.g., *…* I try to solve the problem directly [e.g., have a conversation]). The predetermined if‐then plans were chosen based on latest recommendations for research and practice on planning in health context (Hagger & Luszczynska, 2014; Kaluza, 2018). Apprentices were asked to select two of the nine predetermined if‐then plans that they would use to reduce their occupational perceive stress level on a stress day at apprenticeship site. These preselected if‐then plans formed the basis for the JITPI.

**FIGURE 1:**
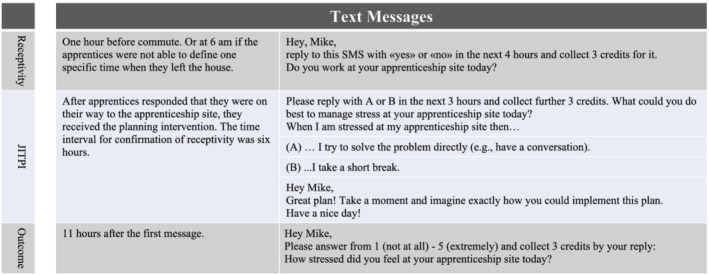
Just‐in‐time planning intervention (ad hoc translated from German)

In the following 16 weeks, the intervention (A) was delivered via short text messages on one of the individually indicated stress days (see Figure 1). The intervention included three steps. First, apprentices received a short and personalized text message that included the assessment of the state of receptivity on the individually indicated stressful day 1 h before the apprentices left their home to go to their apprenticeship site.

In case the apprentices were not able to define one specific time when they left the house, for example, due to shift work, the intervention was delivered at 6 a.m. After apprentices responded that they were on their way to the apprenticeship site, they received the planning intervention. The time interval for confirmation of receptivity was 6 h. Nonresponding participants (within this time period) did not receive the subsequent messages of the planning intervention. The planning intervention comprised a text message to choose one of the two predetermined if‐then plans to practice a stress reduction strategy to reduce the perceived occupational stress level at their apprenticeship site. Further, participants got another text message prompting to visualize the chosen plan. Afterwards, participants received a personalized text message 11 h after the first message to measure their perceived occupational stress level (see Figure 1). The control condition (B) got the same text messages except for the planning intervention.

In order to obtain a maximum sample size for this crossover trial, the state of receptivity was assessed in as many weeks as possible until a participant was receptive twice.^1^ The allocation to the two conditions A and B was randomized. Further, there was a washout period of at least 2 weeks between A and B as well as B and A, respectively (see supporting information S1).

### Participants

The sample size was calculated with the use of G*Power program (Faul et al., 2007). The needed sample size derived from the assumption of a medium effect (*d* = .40). The estimation of the effect size was based on Scholz et al. (2009) plus Gallo et al. (2012). Both studies used implementation intentions in the context of psychological stress (Scholz et al., 2009) and the regulations of emotions (Gallo et al., 2012). To detect differences between the two conditions A and B at *p* < .05 with a power of 1‐*β*, a total of 199 participants are required. With an estimated dropout rate of 30% (60 participants), 259 apprentices were needed to be enrolled in the study.

Overall, 2635 adolescents applied to attend for the ready4life study. Inclusion criteria for the present study were (1) the availability of a mobile phone, (2) being 16 years or older, and (3) the indication of at least 1 day per week that is perceived as stressful at the apprenticeship site. A total of 1681 apprentices registered for the JITPI. Of these, 386 apprentices were eligible and took part in the JITPI (see supporting information S2). The participants had a mean age of *M* = 17.54 (*SD* = 1.92), and 276 of the 386 participants were women (71.50%).

All participants were able to earn credit points for their participation during the study. With these points, they could purchase a selection of incentives (e.g., a voucher for an adventure park). The presented study was registered at Current Controlled Trials ISRCTN 12865220, assigned August 3, 2017. The study protocol was further approved by the Ethics Committee of the first author's institution (date of approval: September 26, 2016). All participants attended voluntarily, signed an informed consent, and were treated in accordance to the standards of the Declaration of Helsinki (World Medical Organization, 1996).

### Measures

The following instruments were included in the questionnaires. All item examples are translations from German. Table 1 shows the means and standard deviations of all measures.

**TABLE 1:** Descriptives of the variables of the model

Participants																			
Variables	*N* = 386						Women (*n* = 276)						Men (*n* = 110)						Range
	BA		CA (*n* = 335)		CB (*n* = 303)		BA		CA (*n* = 238)		CB (n = 218)		BA		CA (*n* = 97)		CB (*n* = 85)		
	*M*	*SD*	*M*	*SD*	*M*	*SD*	*M*	*SD*	*M*	*SD*	*M*	*SD*	*M*	*SD*	*M*	*SD*	*M*	*SD*	
Vigor	3.79	1.14					3.76	1.16					3.85	1.09					0–7
Exhaustion	3.35	1.14					3.41	1.16					3.21	1.07					0–7
Age	17.54	1.92					17.60	2.00					17.38	1.80					16–25
Occupational stress			1.53	1.22	1.64	1.20			1.60	1.24	1.72	1.25			1.36	1.14	1.46	1.01	0–4

*Note*: Condition is coded: 0 = Condition B (control); 1 = Condition A (intervention).

Abbreviations: BA, baseline; CA, condition; CB, condition B.

**Vigor** (Schaufeli et al., 2006) was measured with two items (Cronbach's *α*_t1_ = .74). An item example is “When I get up in the morning, I feel like going to work.” The items were answered on a 6‐point Likert scale ranging from “never” (0) to “always” (5).

**Exhaustion** (Melamed et al., 1992) was assessed with three items (Cronbach's *α*_t1_ = .78). The item stem “How often do you feel at work as described?” was followed by items like “I feel like my batteries are dead.”. The participants were assigned to complete the items on a 6‐point Likert scale ranging from “never” (0) to “very often” (5).

The measurement of the outcome perceived **occupational stress** (self‐developed) took place 11 h after the state of receptivity for both conditions. The item “How stressed did you feel at work today?” was sent by a text message. Participants could response on a 5‐point Likert scale from 0 “not at all” to 4 “extremely” by a text message.

The following control variables were allocated: (1) general perceived stress level, (2) the most stressful day of the week for the participants at their apprenticeship site, (3) state of receptivity, (4) domain‐specific self‐efficacy (“I am confident that I can cope better with stress at the apprenticeship site”. The item could be answered on a 5‐point Likert scale ranging from “not true at all” [1] to “very true” [5]; Sniehotta et al., 2005; Scholz et al., 2008), (5) age, and (6) gender. For parsimony, the unadjusted analyses are reported in this article. The adjusted analyses with the control variables are shown in the supporting information S3.

### Data analysis

Given the fact of the AB/BA design and the within‐subject design, it is important to take the nesting of subjects within clusters, plus the nesting of repeated measurements within subjects into account; therefore, multilevel modeling was used (Brown, 1980; Jones & Kenward, 2015; Moerbeek, 2020; Senn, 2002). The association between the condition and the outcome variable (occupational stress) is nested in different levels: cluster and participants (Goldstein, 2010; Hox et al., 2018; Raudenbush & Bryk, 2002; Snijders & Bosker, 2012). Participants of the same cluster receive the same treatment condition. Due to the crossover design, the clusters are randomized to the two conditions A and B but in separate periods, resulting in the formation of two cluster periods (Moerbeek, 2020). For the analyses, the between‐person (Level 2) predictors (i.e., vigor) were grand‐mean centered at the sample mean. To analyze the hypotheses, linear mixed models with a maximal random effects structure were specified (Barr et al., 2013). In the event of nonconvergence, the random effects structure was successively reduced until convergence was met. Due to the crossover design the analyzed multilevel model for hypotheses 1 is as follows:

$$\gamma_{\mathrm{ij}}=\beta_{0}+\left(\beta_{1}+\upsilon_{\text{1j}}\right)*x_{\text{1ij}}+\left(\beta_{2}+\upsilon_{\text{2j}}\right)*x_{\text{2ij}}+\beta_{3}*\left(x_{\text{1ij}}*x_{\text{2ij}}\right)+\upsilon_{j}+{\text{𝑒}}_{\mathrm{ij}}$$

*y*_ij_ is the outcome of the participant (occupational stress) *i* and *j* = 0,1 the total number of clusters. The predictor *x*_*1ij*_ indicates the time point of measurement and is coded with 0 for the first time point and 1 for the second. The condition (*x*_*2ij*_) is given the persons *i* within the cluster *j* and is equated to 0 for the control and 1 for the intervention condition. The interaction term of condition and time point is described as *x*_*1ij*_ *x_*2ij*_. *υ*_1j_ and *υ*_2j_ are each the residuum of the random effect. *υ*_*j*_ is the residuum of the cluster and *𝑒*_*ij*_ the residuum of participant *i*. *β*_*0*_ is the intercept, and *β*_*1*,_ *β*_*2*,_ and *β*_*3*_ are the overall mean outcome in the first time period for the control condition (B) and the treatment condition (A), respectively. The extended models for the separated analyses of the hypotheses 2 and 3 included the predictor vigor (hypothesis 2) or exhaustion (hypothesis 3) with the interaction term (vigor or exhaustion multiplied with condition) to the equation. The predictor is indicated with *x*_*3ij*_, and the interaction term is shown with *x*_*1ij*_ *x_*3ij;*_ *β*_*4*,_ and *β*_*5*_ are the overall mean outcome in the first time period for the control condition (B) and the treatment condition (A), respectively. The model is established as follows:

$$\gamma_{\mathrm{ij}}=\beta_{0}+\left(\beta_{1}+\upsilon_{\text{1j}}\right)*x_{\text{1ij}}+\left(\beta_{2}+\upsilon_{\text{2j}}\right)*x_{\text{2ij}}+\beta_{3}*x_{\text{3ij}}+\beta_{4}*\left(x_{\text{1ij}}*x_{\text{2ij}}\right)+\beta_{5}*\left(x_{\text{1j}}*x_{\text{3ij}}\right)+\upsilon_{j}+{\text{𝑒}}_{\mathrm{ij}}$$

All analyses were conducted in SPSS 26, with a probability level of *p* = .05. Sensitivity analyses for covariates were conducted for all models. According to the recommendation from York (2018), only those covariates that significantly correlate with the outcome variable (occupational stress) were included to test the most parsimonious model. The data of this study are available from the corresponding author upon reasonable request.

## RESULTS

### Descriptives

Table 1 shows the descriptive statistics for the main variables. Most of the participants identified one specific stress day per week at the apprenticeship site (66.2%). Participants reported medium scores of vigor (*M* = 3.79, *SD* = 1.14) and exhaustion (*M* = 3.35, *SD* = 1.14). Regarding the measured occupational stress level during the intervention period, apprentices reported similar stress level in both conditions A and B (*M*_*ConditionA*_ = 1.53, *SD* = 1.22; *M*_*ConditionB*_ *=* 1.64, *SD =* 1.20; *t*[636] = 1.18; *p* = .240). Young women reported a slightly higher perceived occupational stress level in both conditions as compared with young men (women: *M*_*Condition*A_ = 1.60 *SD* = 1.24; *M*_*Conditiontt*B_ = 1.72, *SD* = 1.25. men: *M*_*Conditiont*A_ = 1.36 *SD* = 1.14; *M*_*Conditiont*B_ = 1.46, *SD* = 1.01). However, the difference in condition A and in condition B was not significant. Table 2 shows the intercorrelations between all variables. As can be seen perceived, occupational stress was related with most variables measured at Baseline, for example vigor (*r* = −.11) and exhaustion (*r* = .21).

**TABLE 2:** Intercorrelations of the main variables and their interactions

Variables		1	2	3	4	5	6	7	8	9	10
1.	Stress										
2.	Condition	−.047									
3.	Time	−.063	−0.041								
4.	Condition*time	−.066	.**561** ^**^	**.561** ^**^							
5.	Exhaustion	**.205** ^**^	.000	.000	.019						
6.	Vigor	**−.111** ^**^	.000	.000	−.014	**−.644** ^**^					
7.	Condition*Vigor	**−.102** ^**^	.000	−.017	−.020	**−.455** ^**^	**.707** ^**^				
8.	Condition*Exhaustion	**.162** ^**^	.000	.022	.026	**.707** ^**^	**−.455** ^**^	**−.644** ^**^			
9.	Age	−.022	.000	0.000	−.047	**.139** ^**^	.021	.015	**.099** ^**^		
10.	Sex	**.093** ^*^	.000	.000	.032	**.078** ^*^	−.037	−.026	.055	.052	

* *p* < .05.

** *p* < .01.

*** *p* < .001.

The significant values are bold to better identify them.

### Results of the hypotheses

Hypothesis 1 stated that on days when apprentices receive the JITPI intervention, apprentices report lower levels of stress at apprenticeship site as compared with days without the JITPI. Complete statistical results for the general linear mixed model testing hypothesis 1 are presented in Table 3. For the fixed effects, the results on intercept, condition, time, and the interaction term of condition and time are given. The intercept describes that an average apprentice had a stress value of 1.72 (range 0 to 4) at the first measurement point. No significant effect of the condition A (planning intervention: *b* = −11, *p* = .401) was found in contrast to the average level of occupational stress in condition B, meaning conditions A and B did not differ. Time was not significantly related to occupational stress; thus, occupational stress did not change significantly over the two measurement points. Additionally, the interaction term of condition and time did not show any significant effect on occupational stress. Also, the random effects of the intercept or the residual variance were not significant. Hence, there were no interindividual differences between the apprentices and there were no significant nonsystematic influences that could not be explained by the model (Bolger & Laurenceau, 2013). Further, there were no discrepancies between the predicted and real values (Bolger & Laurenceau, 2013). Since the autocorrelation was not significant, no within‐person dependence of the residuals could be detected. This implies that person‐specific variables not included in the model had no effect on occupational stress (Bolger & Laurenceau, 2013).

**TABLE 3:** Parameter estimates for linear mixed model of hypothesis 1

					*CI*_*95*_	
Fixed effects	Estimate	(*SE*)	*t*	*p*	Lower	Upper
Intercept	**1.72**	(.09)	18.23	<.001	1.53	1.90
Condition	−.11	(.13)	−.84	.401	−.36	.14
Time	−.15	(.14)	−1.13	.259	−.42	.11
Condition*Time	.00	(.21)	.01	.990	−.41	.41

					*CI*_*95*_	
Random effects ([co]‐variance)	Estimate	(*SE*)	*z*	*p*	Lower	Upper
Level 2 (between‐person)^a^						
Intercept	.56	(83.80)	.01	.995	0.00	8.55
Condition	‐	‐	‐	‐	‐	‐
Time	.14	(.16)	.88	.378	.02	1.29
Level 1 (within‐person)						
Residual	.81	(83.80)	.01	.992	0.00	2.92
Autocorrelation	−.23	(126.46)	−.00	.999	−1.00	1.00

*Note*: *N* = 386. All *p*‐values are two‐tailed; condition is coded: 0 = Condition B (control); 1 = Condition A (intervention); time is coded: 0 = stress day 1; 1 = stress day 2.

Abbreviations: CI, confidence interval; SE, standard error.

^a^ The model did not converge with a maximum random effects structure, so according to Barr et al. (2013), condition was removed from the model.

The significant values are bold to better identify them.

Hypothesis 2 predicts that apprentices with lower levels of occupational vigor benefit from the JITPI to a higher degree as compared with apprentices with higher levels of occupational vigor. Complete statistical results for testing hypothesis 2 are presented in Table 4. As can be seen by the intercept of the fixed effects, the average level of perceived occupational stress on the first condition was 1.72 on a scale from 0 to 4 (*p* < .001). All variables including vigor and the interaction term of vigor and time had no significant effect on perceived occupational stress. Thus, no moderation effect could be found. Further, the random effects did not show significant effects.

**TABLE 4:** Parameter estimates for linear mixed model of hypothesis 2

					*CI*_*95*_	
Fixed effects	Estimate	(*SE*)	*t*	*p*	Lower	Upper
Intercept	**1.72**	(.09)	18.21	<.001	1.53	1.91
Condition	−.10	(.13)	−.78	.437	−.35	.15
Time	−.15	(.14)	−1.12	.264	−.42	.12
Condition*Time	−.02	(.21)	−.08	.936	−.42	.39
Vigor	−.07	(.06)	−1.19	.234	−.19	.05
Vigor*Condition	−.08	(.07)	−1.09	.277	−.22	.06

					*CI*_*95*_	
Random effects ([co]‐variance)	Estimate	(*SE*)	*z*	*p*	Lower	Upper
Level 2 (between‐person)^a^						
Intercept	.56	(106.67)	.01	.996	0.00	1.95
Condition	‐	‐	‐	‐	‐	‐
Time	.09	(.16)	.57	.567	.00	2.76
Level 1 (within‐person)						
Residual	.82	(106.67)	.01	.994	0.00	1.09
Autocorrelation	−.25	(163.25)	−.00	.999	−1.00	1.00

*Note*: *N* = 386. All *p*‐values are two‐tailed; condition is coded: 0 = Condition B (control); 1 = Condition A (intervention); time is coded: 0 = stress day 1; 1 = stress day 2; vigor is coded: 0 = never; 5 = always.

Abbreviations: CI, confidence interval; SE, standard error.

^a^ The model did not converge with a maximum random effects structure, so according to Barr et al. (2013) condition was removed from the model.

The significant values are bold to better identify them.

Hypothesis 3 postulates that apprentices with higher levels of work‐related exhaustion benefit from the JITPI to a higher degree compared with adolescents with lower levels of work‐related exhaustion. In Table 5, complete statistical results for testing hypothesis 3 are shown. The intercept of the fixed effects (the average level of stress on the first condition) was 1.73 on a scale from 0 to 4 (*p* < .001). Moreover, exhaustion had a positive significant effect on stress (*b* = .20, *p* < .05) indicating that trainees with high exhaustion levels were more stressed than trainees with low exhaustion levels. All other variables did not reach statistical significance. The random effects did not show significant effects.

**TABLE 5:** Parameter estimates for linear mixed model of hypothesis 3

					*CI*_*95*_	
Fixed effects (intercept, slopes)	Estimate	(*SE*)	*t*	*p*	Lower	Upper
Intercept	**1.73**	(.09)	18.62	<.001	1.54	1.91
Condition	−.10	(.13)	−.76	.447	−.35	.15
Time	−.15	(.13)	−1.11	.268	−.41	.11
Condition*Time	−.04	(.20)	−.18	.856	−.44	.36
Exhaustion	**.20**	(.06)	3.33	.001	.08	.31
Exhaustion*Condition	.04	(.07)	.55	.582	−.11	.19

					*CI*_*95*_	
Random effects ([co]‐variance)	Estimate	(*SE*)	*z*	*p*	Lower	Upper
Level 2 (between‐person)						
Intercept	.60	(94.16)	.01	,995	1.09	3.26
Condition	.06	(.16)	.38	.704	.00	10.43
Time	.08	(.16)	.47	.636	.00	4.72
Level 1 (within‐person)						
Residual	.73	(94.15)	.01	.994	8.00	6.21
Autocorrelation	−.39	(179.28)	−.00	.998	−1.00	1.00

*Note*: *N* = 386. All p‐values are two‐tailed; condition is coded: 0 = Condition B (control); 1 = Condition A (intervention); time is coded: 0 = stress day 1; 1 = stress day 2; exhaustion is coded: 0 = never; 5 = very often.

Abbreviations: CI, confidence interval; SE, standard error.

The significant values are bold to better identify them.

Taken together, all three hypotheses could not be confirmed.

## DISCUSSION

The present study was designed to test the effectiveness of a JITPI to reduce perceived occupational stress in apprentices. Contrary to our hypotheses, the findings demonstrated that perceived occupational stress could not be reduced by the planning intervention condition in this study. Further, no moderating effects of the intervention effect by vigor and exhaustion emerged. It has to be concluded that the JITPI was not effective to reduce perceived occupational stress.

One explanation might be the overall low stress level of the sample. Given the unexpected low perceived occupational stress, the probability was very low that it could be reduced even further by the JITPI. Therefore, one can assume a selection effect in that highly stressed apprentices did not participate in the study. Thus, future studies should focus on specific vulnerable groups with higher levels of perceived occupational stress or a high‐risk stress environment at the apprentices' sites by identifying in advance which groups of apprentices are most affected by stress and by specifying the inclusion criteria of the study in more detail.

Another reason for the findings might be that apprentices perceived no stress at the apprenticeship site on their indicated stress day as this was defined several weeks before the measurement. Future studies should combine the JITPI with mobile and sensing technologies to measure stress in real life situations (Can et al., 2019). This could allow JITPIs to be used in a timely manner based on objective data (e.g., heart rate variability, Goel et al., 2021).

A third explanation for the null finding might be that perceived occupational stress was assessed 11 h after the JITPI, mostly in the evening. Possibly, the participants perceived occupational stress differently in the evening than during work or had already coped successfully when the stress was assessed. Stress responses may depend on the daytime (Yamanaka et al., 2019). Therefore, it could be assumed that the participants were possibly more relaxed in the evening independent from the intervention. A measure of occupational stress during work or right after an experienced stress event might have revealed an intervention effect. As already mentioned, a device‐based measurement of stress is recommended for future studies, for example, by measuring the heart rate (Kim et al., 2018). In case of an objectively assessed physical stress response, a reminder to apply the if‐then plans could be sent from the system via SMS and the apprentices can immediately start to apply the plans.

A fourth potential reason why the intervention showed no effect on perceived occupational stress might be based in the conduction of the JITPI. First, it was not asked whether participants were motivated to use stress reduction strategies on that very day, so planning may not have matched participants' motivational status. Because it can be assumed that motivated participants are more likely to implement the volitional strategy of planning (e.g., Gollwitzer, 1999; Schwarzer, 2008), future studies should assess the motivational status on days planning interventions are offered. Additionally, participants had to select two of nine plans provided by the researcher in the baseline. Thus, the participants were not involved in the development of the plans. In addition, the plans were formulated in a broad sense that may not have always been the best fit for the individual or the apprenticeship site. Even though Armitage (2008) has shown the effectiveness of predefined plans, self‐defined plans might be more content‐specific and relevant for people. Participants might be more motivated and committed to their plans in comparison with participants that had to select between predefined plans (Sniehotta, 2009). Since apprenticeships are very heterogeneous, future studies should enable individualized plans as it might be the case that the used plans in this study did not fully cover the apprenticeship circumstances. Furthermore, more research is also suggested about the optimum number of plans (Wiedemann et al., 2011) required to attain stress reduction that is still feasible in a JITPI.

Another aspect that needs to be mentioned is that according to Skår et al. (2011), 20%–40% of participants do not act upon their plans. Due to the fact that not all participants responded to the JITPI, it might be the case that participants of the current study possibly did not adhere to the chosen plans. Since the commitment and other user engagement factors of the apprentices were not assessed, no statement can be made about adherence to the plans. Future studies should assess whether the plans have been adhered (Keller et al., 2017; Fleig et al., 2017) or should monitor the adherence of the plans more closely, for example, with a daily diary assessment (Berli et al., 2018). Furthermore, it should be tested whether the planned behavior is associated with or mediates potential intervention effects on perceived occupational stress.

Overall, the findings are also in line with other planning inventions which indicated that planning has a very small effect on youth's health behavior like physical activity (Koka & Hagger, 2017; Luszczynska et al., 2016). Perhaps this phenomenon is also present in apprentices in the context of a stress intervention. Since the present JITPI with planning on stress reduction has been conducted for the first time in adolescents and young adults, more research with participants experienced with high occupational stress is needed. Before planning further JITPIs, we suggest to also observe vulnerable groups in more detail by, for example, using daily diary assessment (Berli et al., 2018) to gain more insight about the right moment to intervene or about already applied successful coping strategies at the right moment to handle stressful situations.

### Strength and limitations

A strength of the present study is that the JITPI enables brief interventions in daily life (Hardeman et al., 2019). Yet, some limitations also need to be kept in mind: First, although the crossover design applied has all the advantages of a within‐person design (e.g., it avoids problems of comparability of study and control group because each participant is his/her own control), carryover effects might have confounded parts of the intervention effects (Moerbeek, 2020). However, since there was a washout period of at least 2 weeks, the effects could be small. Second, the query of the state of receptivity only asked whether the person is working at the apprenticeship site at this day. But it was not questioned if a stress event was expected. With a more precise state of receptivity query or more precise fitting delivery of the intervention, including additional parameters (i.e., general resource, mood, content, and media employed for delivery or the inclusion of objective measurement that indicates when someone is stressed; Hardeman et al., 2019), it is likely to achieve a better fit of the intervention for the participants. Also, it should be clarified beforehand whether occupational stress happens daily or less frequently. In the case of daily occurrence, it can be assumed that the apprentices perceive the stress differently and react to it differently. Further, investigations should therefore be conducted to determine exactly when and why a person needs JITPIs regarding occupational stress. A further limitation is that a JITPI is maybe not sufficient for the change of the perceived stress level among adolescents and young adults. Therefore, future study should use a more interactive mobile app‐based digital intervention, for example, a just‐in time adaptive intervention (JITAI; Hardeman et al., 2019). JITAIs have the capacity to support people when they are in situations to engage in unhealthy behaviors and can also suggest alternative health behaviors. By incorporating digital technology, data can be collected in real time and allow taking the user's environment and/or their current mood and emotions into account. With this knowledge, an immediate and automated support for behavioral changes is possible (Hardeman et al., 2019).

## CONCLUSION

Our study provides insight into JITPI to reduce perceived occupational stress of apprentices. Even though the examined JITPI did not have an effect on the reduction of occupational stress in this setting, one should not reject JITPIs as a strategy to regulate occupational stress. Instead, future studies should particularly focus on increasing the reach of JITPIs, for example, by providing support in a high‐risk environment or vulnerable groups.

## CONFLICT OF INTEREST

The authors declared that they had no conflicts of interest with respect to their authorship or the publication of this article

## ETHICS STATEMENT

The presented study was registered at Current Controlled Trials ISRCTN 12865220, assigned August 3, 2017, and was further approved by the Ethics Committee of the first author's institution (date of approval: September 26, 2016). As mentioned in the manuscript, all participants attended voluntarily, signed an informed consent, and were treated in accordance to the standards of the Declaration of Helsinki (World Medical Organization, 1996).

## AUTHOR CONTRIBUTION

Hereby, we confirm that all authors have read and followed the instructions for authors, that the submitted manuscript is the intellectual property of the authors, that all authors have contributed substantially, seen, and approved the current version of the manuscript, that there is no conflict of interests, that it has not been published before, that it does not contain data that are currently submitted or published elsewhere, that we have full control of all primary data, and that we agree to allow the journal to review our data if requested.

## FUNDING INFORMATION

The first author was funded by the Swiss National Science Foundation, SNSF Grant number: 100019_169781/1. The sponsor had no influence on the study design, the data collection, or analyses.

## Supporting information


**Data S1.** Supporting Information

## Data Availability

If the manuscript will be accepted, the data will be made available.

## ENDNOTE

^1^
Here we deviate from the study protocol. Originally, six measurements point were planned. Since the response rate in the field was very low, the design was adapted so that only two measurements points per person were collected in order to keep the participants burden low in the overall project “ready4life.”
