# Supplementary material for: One SMS a day keeps the stress away? A just‐in‐time planning intervention to reduce occupational stress among apprentices
Source: Appl Psychol Health Well Being. 2022 Jan 20;14(4):1389–407. doi: 10.1111/aphw.12340 (PMC9786884; doi:10.1111/aphw.12340)
Supplement: Supplementary file 1 — Data S1. Supporting Information [file APHW-14-1389-s001.docx]

**Randomizationn**

**Study participants**

**ConditionA**

**Washout**

**period of min. 2 weeks**

**Condition B**

**Condition B**

**Condition A**

**Period 1**

**Period 2**

*Figure A1*. Study design

Occupational stress in CA assessed (*n*=148)

Occupational stress in CB assessed (*n*=150)

Occupational stress in CA assessed

(*n*=187)

Occupational stress in CB assessed

(*n*=153)

Randomization:

TB –TA (*n*=185)

Randomization:

TA – TB (*n*=201)

Participants of the *module 1* intervention program

(*N*=1681)

Did not meet the age inclusion criteria (*n*=46)

No stress at the apprenticeship site (*n*=663)

No preparation of the if-then-plan (*n*=61)

No stress event or no participation of the JITAI

(*n*=516)

[implausible values](https://www.linguee.com/english-german/translation/implausible+values.html) (*n*=9)

Participants of the JITAI occupational stress program

(*n*=972)

Participants of the JITAI occupational stress program and data on preferred if-then-plan (*n*=386)

Washout phase

*Figure A2.* Assessment for eligibility.

*Note*. CA: Condition A, planning intervention; CB: Condition B, control condition.

|  |  |  |  |  | *CI_95_* | |
| --- | --- | --- | --- | --- | --- | --- |
| Fixed effects | Estimate | *(SE)* | *t* | *p* | Lower | Upper |
| Intercept | **1.85** | .10 | 17.69 | .000 | 1.64 | 2.05 |
| Condition | -.10 | .13 | -.76 | .448 | -.35 | .153 |
| Time | -.15 | .13 | -1.09 | .28 | -.41 | .12 |
| Condition*Time | -.03 | .20 | -.145 | .885 | -.43 | .37 |
| Most stressful day | -.23 | .11 | -2.12 | .035 | -.43 | -.02 |
| Self-efficacy | **-.12** | .06 | -2.15 | .032 | -.23 | -.01 |
| General perceived stress level | .04 | .02 | 1.88 | .061 | .00 | .08 |
| Sex | -.17 | .12 | -1.49 | .138 | -.40 | .06 |
|  |  |  |  |  | *CI_95_* | |
| Random effects ([co]-variance) | Estimate | (*SE*) | *z* | *p* | Lower | Upper |
| Level 2 (between-person) ^A^ |  |  |  |  |  |  |
| Intercept | .61 | 75.25 | .008 | .994 | 2.12 | 1.74 |
| Condition | .04 | .16 | .271 | .786 | 3.05 | 57.89 |
| Time | .07 | .16 | .428 | .669 | .00 | 6.55 |
| Level 1 (within-person) |  |  |  |  |  |  |
| Residual | .73 | 75.25 | .010 | .992 | 3.42 | 1.54 |
| Autocorrelation | -.38 | 143.66 | -.003 | .998 | -1.00 | 1.00 |

*Table A1*. Parameter Estimates with covariates for Linear Mixed Model of Hypothesis 1

*Note. N* =386

All *p*-values are two-tailed; Condition is coded: 0 = Condition B (control); 1 = Condition A (intervention); Time is coded: 0 = stress day 1; 1 = stress day 2; Most stressful day is coded: 0 = same stress day for both measurement; 1 = different stress day for both measurement points; self-efficacy is coded: 0 = not true at all; 4 = very true; general perceived stress level is coded: 0 = never; 4 = very often; sex is coded: 0 = men; 1 = women. SE = Standard Error; CI = Confidence Interval.

*Table A2.* Parameter Estimates with covariates for Linear Mixed Model of Hypothesis 2

*Note. N* =386

All *p*-values are two-tailed; Condition is coded: 0 = Condition B (control); 1 = Condition A (intervention); Time is coded: 0 = stress day 1; 1 = stress day 2; Vigor is coded: 0 = never; 5 = always; Most stressful day is coded: 0 = same stress day for both measurement; 1 = different stress day for both measurement points; self-efficacy is coded: 0 = not true at all; 4 = very true; general perceived stress level is coded: 0 = never; 4 = very often; sex is coded: 0 = men; 1 = women. SE = Standard Error; CI = Confidence Interval.

|  |  |  |  |  | *CI_95_* | |
| --- | --- | --- | --- | --- | --- | --- |
| Fixed effects | Estimate | *(SE)* | *t* | *p* | Lower | Upper |
| Intercept | **1.85** | .105 | 17.63 | .000 | 1.64 | 2.05 |
| Condition | -.09 | .13 | -.70 | .485 | -.34 | .16 |
| Time | -.14 | .13 | -1.08 | .282 | -.41 | .12 |
| Condition*Time | -.04 | .20 | -.20 | .838 | -.44 | .36 |
| Vigor | -.02 | .06 | -.28 | .782 | -.14 | .10 |
| Vigor*Condition | -.09 | .07 | -1.17 | .245 | -.23 | .06 |
| Most stressful day | **-.22** | .11 | -2.09 | .037 | -.43 | -.01 |
| Self-efficacy | -.11 | .06 | -1.85 | .065 | -.22 | .01 |
| General perceived stress level | .03 | .02 | 1.57 | .116 | -.01 | .07 |
| Sex | -.18 | .12 | -1.57 | .117 | -.41 | .05 |
|  |  |  |  |  | *CI_95_* | |
| Random effects ([co]-variance) | Estimate | (*SE*) | *z* | *p* | Lower | Upper |
| Level 2 (between-person) |  |  |  |  |  |  |
| Intercept | .62 | 78.38 | .008 | .994 | 3.79 | 1.00 |
| Condition | .03 | .16 | .19 | .85 | -7.30 | 1124.99 |
| Time | .04 | .16 | .26 | .80 | -2.05 | 80.188 |
| Level 1 (within-person) |  |  |  |  |  |  |
| Residual | .73 | 78.38 | .01 | .993 | -1.89 | 2.81 |
| Autocorrelation | -.40 | 150.43 | .00 | .998 | -1.00 | 1.00 |

.

|  |  |  |  |  | *CI_95_* | |
| --- | --- | --- | --- | --- | --- | --- |
| Fixed effects (intercept. slopes) | Estimate | *(SE)* | *t* | *p* | Lower | Upper |
| Intercept | **1.85** | .10 | 17.61 | .000 | 1.64 | 2.05 |
| Condition | -.09 | .13 | -.68 | .495 | -.34 | .16 |
| Time | -.14 | .13 | -1.04 | .297 | -.40 | .12 |
| Condition*Time | -.06 | .20 | -.28 | .78 | -.45 | .34 |
| Exhaustion | **.15** | .06 | 2.38 | .018 | .03 | .28 |
| Exhaustion*Condition | .05 | .07 | .60 | .546 | -.10 | .19 |
| Most stressful day | -.19 | .11 | -1.84 | .067 | -.40 | .02 |
| Self-efficacy | -.08 | .06 | -1.44 | .150 | -.19 | .03 |
| General perceived stress level | .013 | .02 | .63 | .531 | -.03 | .05 |
| Sex | -.19 | .12 | -1.68 | .094 | -.42 | .03 |
|  |  |  |  |  | *CI_95_^d^* | |
| Random effects ([co]-variance) | Estimate | (*SE*) | *z* | *p* | Lower | Upper |
| Level 2 (between-person) |  |  |  |  |  |  |
| Intercept | .61 | 91.58 | .01 | .995 | -1.68 | 2.22 |
| Condition | .03 | .16 | .20 | .841 | -1.80 | 545.78 |
| Time | .029 | .16 | .18 | .854 | -7.02 | 1214.00 |
| Level 1 (within-person) |  |  |  |  |  |  |
| Residual | .74 | 91.58 | .01 | .994 | -5.51 | 9.79 |
| Autocorrelation | -.41 | 176.33 | .00 | .998 | -1.00 | 1.00 |

*Table A3.* Parameter Estimates with covariates for Linear Mixed Model of Hypothesis 3

*Note. N* =386

All *p*-values are two-tailed; Condition is coded: 0 = Condition B (control); 1 = Condition A (intervention); Time is coded: 0 = stress day 1; 1 = stress day 2; Most stressful day is coded: 0 = same stress day for both measurement; 1 = different stress day for both measurement points; self-efficacy is coded: 0 = not true at all; 4 = very true; general perceived stress level is coded: 0 = never; 4 = very often; sex is coded: 0 = men; 1 = women. SE = Standard Error; CI = Confidence Interval.
